# Respiratory viral coinfection in a birth cohort of infants in rural Nepal

**DOI:** 10.1111/irv.12775

**Published:** 2020-06-22

**Authors:** Anne Emanuels, Stephen E. Hawes, Kira L. Newman, Emily T. Martin, Janet A. Englund, James M. Tielsch, Jane Kuypers, Subarna K. Khatry, Steven C. LeClerq, Joanne Katz, Helen Y. Chu

**Affiliations:** ^1^ Department of Epidemiology University of Washington Seattle WA USA; ^2^ Department of Laboratory Medicine University of Washington Seattle WA USA; ^3^ University of Michigan Ann Arbor MI USA; ^4^ Seattle Children’s Hospital Seattle WA USA; ^5^ Department of Global Health George Washington University Milken Institute School of Public Health Washington DC USA; ^6^ Department of International Health Johns Hopkins Bloomberg School of Public Health Baltimore MD USA; ^7^ Nepal Nutrition Intervention Project – Sarlahi (NNIPS) Kathmandu Nepal

**Keywords:** coinfection, epidemiology, global health, human influenza, human respiratory syncytial virus, respiratory infections

## Abstract

**Background:**

Acute respiratory illnesses are a leading cause of global morbidity and mortality in children. Coinfection with multiple respiratory viruses is common. Although the effects of each virus have been studied individually, the impacts of coinfection on disease severity are less understood.

**Methods:**

A secondary analysis was performed of a maternal influenza vaccine trial conducted between 2011 and 2014 in Nepal. Prospective weekly household‐based active surveillance of infants was conducted from birth to 180 days of age. Mid‐nasal swabs were collected and tested for respiratory syncytial virus (RSV), rhinovirus, influenza, human metapneumovirus (HMPV), coronavirus, parainfluenza (HPIV), and bocavirus by RT‐PCR. Coinfection was defined as the presence of two or more respiratory viruses detected as part of the same illness episode.

**Results:**

Of 1730 infants with a respiratory illness, 327 (19%) had at least two respiratory viruses detected in their primary illness episode. Of 113 infants with influenza, 23 (20%) had coinfection. Of 214 infants with RSV, 87 (41%) had coinfection. The cohort of infants with coinfection had increased occurrence of fever lasting ≥ 4 days (OR 1.4, 95% CI: 1.1, 2.0), and so did the subset of coinfected infants with influenza (OR 5.8, 95% CI: 1.8, 18.7). Coinfection was not associated with seeking further care (OR 1.1, 95% CI: 0.8, 1.5) or pneumonia (OR 1.2, 95% CI: 0.96, 1.6).

**Conclusion:**

A high proportion of infants had multiple viruses detected. Coinfection was associated with greater odds of fever lasting for four or more days, but not with increased illness severity by other measures.

## 1 INTRODUCTION

Acute lower respiratory tract infections (ALRI), including pneumonia and bronchiolitis, were responsible for an estimated 650 000 deaths of children under five years old in 2016 and continue to be a major cause of infant morbidity and mortality worldwide.^1^ Multiple viruses cause respiratory illness in children, including influenza, respiratory syncytial virus (RSV), rhinovirus, enterovirus, and adenovirus.^2^ Influenza is among the most common etiologies of ALRI episodes and is responsible for an estimated 39 million cases each year among both children and adults. An estimated 30 000‐100 000 children under 5 years old die from influenza each year, with 99% of these occurring in developing countries.^3^ RSV is responsible for over 33 million new cases of childhood ALRI each year, and an estimated 55 000 to 190 000 deaths of children under 5 years old can be attributed to ALRI from RSV alone.^4^ Multiple viruses are often simultaneously detected, though the impact of coinfection is not well‐defined. Further, little research has been done to examine viral coinfection in low resource settings.

Prevention of morbidity and mortality due to respiratory viral disease remains a major goal worldwide. Currently, seasonal influenza vaccines are widely available, and developing an RSV vaccine is among the primary 2020‐2025 goals for GAVI (Global Alliance for Vaccines and Immunization) and the WHO.^5^, ^6^ Understanding the role of coinfections will help inform the relative burden of disease of each respiratory virus and add context to future vaccination intervention and development efforts.

Polymerase chain reaction (PCR) technology has allowed for detection of multiple simultaneous viruses in children with acute respiratory illness.^7^ Before widespread use of molecular diagnostics, concurrent infection by two or more viruses in children under five was considered rare. However, recent studies estimate viral coinfections in 10%‐30% of pediatric patients with ALRI.^8^ A number of previous studies have investigated the relationship between coinfection with multiple viruses and disease severity. These studies have mostly occurred in hospitalized settings in industrialized countries, and findings are discordant on the effect of coinfection on symptom severity and duration.^2^, ^8^

The hypothesis that multiple viral infections can have an additive effect on illness severity is intuitive. A prospective study of hospitalized infants in 2005 found that coinfections of any type were associated with more severe ALRI than single infections in infants under 6 months old.^2^ Other studies confirmed that coinfection resulted in worse outcomes, including higher likelihood of fever,^9^ longer duration of symptoms,^10^ higher rates of admission to the pediatric intensive care unit,^11^ and more severe bronchiolitis.^12^ However, recent viral modeling has indicated that one virus can occupy all of the patient's available cellular mechanisms, inhibiting progress of the other infections. According to this model, competition between two different infections can attenuate illness severity.^13^ Additionally, one infection can trigger an immune response that is protective against the subsequent infections, making coinfections less severe than one virus alone.^14^ By contrast, other reports have found that coinfection did not lead to more severe disease and demonstrate the need for further investigation of the impacts of concurrent infections in infants.^10^, ^15^

As most studies of coinfection have been conducted in hospitalized settings in industrialized countries, little is known about the impact of coinfection on the severity of respiratory viral infection in rural settings. The presence of multiple viral infections may play a role in the severity of these illnesses. The objective of this study is to characterize the frequency and patterns of coinfection in infants in rural Nepal and compare symptom severity in coinfection and singular infection.

## 2 MATERIALS AND METHODS

This is a secondary analysis of data from a prospective, randomized, placebo‐controlled study of the effects of influenza immunization among pregnant women in the Sarlahi District of rural Nepal. Methods and results of this clinical trial have been published and registered with ClinicalTrials.gov (Trial no. NCT01034254).^16^ Between 2011 and 2014, 3693 women were enrolled during pregnancy into a study of maternal influenza immunization. Both women and their infants were monitored via weekly household visits for respiratory illness. Infants enrolled in this study were enrolled in a home visit with an infant birth assessment within 72 hours of birth and followed by weekly visits until they reached 180 days of age. ^17^ Mothers were asked about respiratory symptoms in infants for each day in the past week. Nasal swabs were collected from the infants if they experienced one or more symptoms of respiratory illness in the past week, defined as subjective fever, cough, wheeze, difficult or rapid breathing, or a draining ear. Samples were tested for respiratory viruses by real‐time reverse transcription PCR. ^18^

This secondary study analyzed the subset of infants experiencing their first case of respiratory viral illness. First viral illness was defined as the first symptomatic respiratory illness with a virus detected on the swab. The effect of concurrent viral infections is likely confounded by the relative timing of each infection, as well as the infant's prior illness episodes. To isolate the effects of coinfection from those of prior illnesses, or secondary infections occurring in the weeks following primary infection, only the first swab of the first illness episode was included in this analysis. Monoinfection was defined as a single‐virus respiratory illness. Coinfection was defined as having two or more viruses detected concurrently in the nasal swab. Seven respiratory viruses were included: influenza (types A and B), RSV, rhinovirus (HRV), human metapneumovirus (HMPV), coronavirus (CoV), parainfluenza viruses (HPIV types I‐IV), and bocavirus (BoV). Influenza A and B were combined, as well as HPIV I‐IV. We evaluated the associations between coinfection compared to monoinfection and three pre‐specified outcomes: subjective fever lasting four days or longer, pneumonia, and seeking further medical care. Fever duration was analyzed as a binary outcome to maintain consistency in analysis with other measures of severity. Pneumonia was defined as cough and/or difficulty breathing, combined with wheeze and/or age‐specific tachypnea.^17^ Seeking further medical care was defined as a visit to a hospital, a doctor, or a non‐doctor provider for the primary illness episode in the week preceding the home visit. Non‐doctor providers included visits to local healers and medicine shops. These measures for severe disease were chosen based off of the symptoms and behaviors attributable to respiratory illness observed in this population.

Sociodemographic characteristics and symptoms were compared between infants with coinfection and monoinfection. Continuous variables were described using mean (standard deviation), and binary variables were described using counts and percent of coinfection or monoinfection cases. Two‐sided t tests and chi‐squared tests were used to compare differential distributions of demographic characteristics between infants with coinfection and monoinfection. A variable was determined to differ significantly if the *P*‐value was <.05.

Multivariable logistic regression was used to evaluate the associations between coinfection and risk of each clinical outcome. Separate models compared any coinfection to any monoinfection, coinfection of influenza plus at least one other virus to influenza monoinfection, and coinfection of RSV plus at least one other virus to RSV monoinfection. Influenza and RSV were chosen a priori for additional analysis because of their clinical relevance, their high pathogenicity, and their substantial global burden of disease. Covariates considered as confounders for the associations between coinfection and each clinical severity outcome included infant age in weeks, low birth weight (LBW) defined as weighing <2500 grams at delivery, maternal flu vaccination receipt, and ordinal number of other children <5 years of age in the household. These were adjusted for in the associations between coinfections and each clinical outcome. Odds ratios (ORs) and 95% confidence intervals were reported for each multivariable logistic regression model. A sensitivity analysis was conducted to compare clinical severity in coinfection versus monoinfection after excluding rhinovirus cases in order to separately examine the effects of coinfection among clinically relevant viruses that are less common than rhinovirus.

All analyses were performed using R version 3.4.3 (R Foundation for Statistical Computing) in RStudio Version 1.0.153 (RStudio, Inc). Institutional review board approval for the randomized controlled trial was given by the Johns Hopkins University School of Public Health, Cincinnati Children's Hospital, the Institute of Medicine at Tribhuvan University, and the Nepal Health Research Council.

## 3 RESULTS

Altogether, 1730 (47%) of the 3646 enrolled infants had at least one episode of viral respiratory illness over the course of this study. Among those experiencing their first respiratory viral infection, 1403 (81%) were infected with a single virus and 327 (19%) had at least two respiratory viruses detected simultaneously. Of those with coinfection, 32 (9.8%) had three different viruses detected, and two (0.6%) had four different viruses. Subjects had a mean age of 11 weeks, 54% were male (n = 936), 13% were premature (n = 225), and 20% were born low birth weight (n = 349).

Coinfection status did not differ substantially by sex, whether the infant was premature or low birth weight, the number of rooms in the home, the number of children under 5 years old in the household, or whether the mother smoked (Table 1). Mother's age at delivery was older among mothers of monoinfected infants than among mothers of coinfected infants. Infants with a single infection were more likely to have a latrine in the home (45.2%) than infants with coinfection (38.2%). Among mothers of infants with coinfections, 45.5% had received the flu vaccine compared to 49.5% of monoinfected infants’ mothers receiving the vaccine. Infants with coinfection had a mean age of 12.1 weeks and were an average of 1.3 weeks older than infants with monoinfection. Fever was detected among 77% of infants who tested positive for influenza, while the rate of fever among infants with other viral infections was between 46% for infants with rhinovirus alone and 66% for infants with RSV alone (see Appendix S1).

**Table 1:** Demographic characteristics of infants experiencing their first respiratory viral illness within a maternal influenza trial in rural Nepal between 2011 and 2014 (n = 1730). Variables described using mean (± SD) or n (%)

	Monoinfection (n = 1403)	Coinfection (n = 327)
Maternal and household characteristics		
Mother's age at delivery	23.2 (± 4.8)	22.6 (± 4.5)
Number of children < 5 in Home	1.27 (± 1.30)	1.33 (± 1.20)
Rooms in home	2.75 (± 2.40)	2.67 (± 3.56)
Latrine in home	635 (45.2%)	125 (38.2%)
Mother smokes^a^	56 (4.0%)	10 (3.1%)
Mother received influenza vaccine	695 (49.5%)	149 (45.5%)
Infant characteristics		
Infant age at first illness, weeks	10.8 (± 6.7)	12.1 (± 6.7)
Male	773 (55.1%)	163 (49.8%)
Preterm delivery, <37 weeks	180 (12.8%)	45 (13.6%)
Low birth weight, <2500 g^a^	282 (20.0%)	67 (20.3%)
Year of birth		
2011	439 (31.3%)	105 (32.1%)
2012	631 (45.0%)	155 (47.4%)
2013	333 (23.7%)	67 (20.5%)

^a^ Variables with notable missingness (>5%).

**Figure 1:**
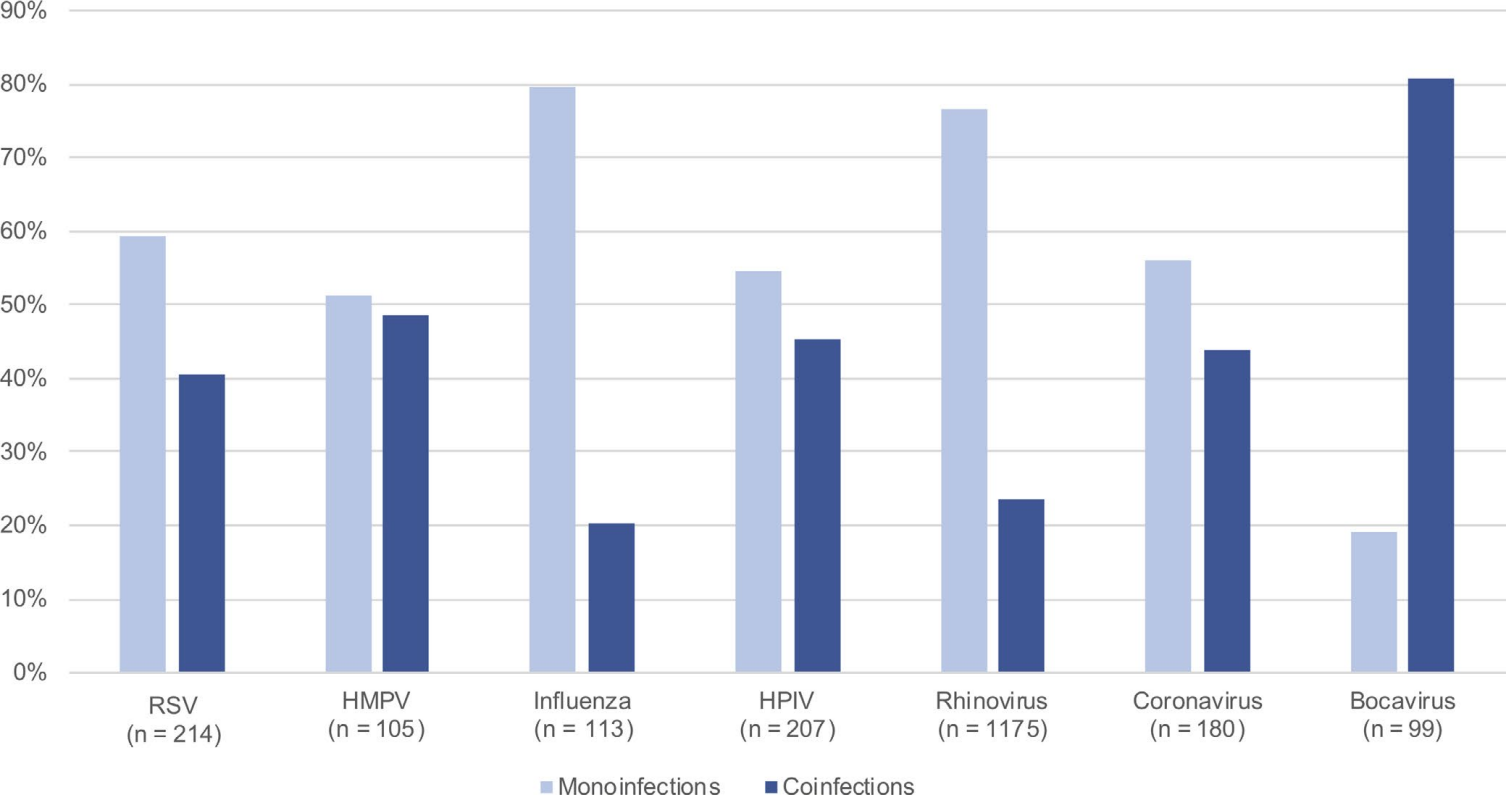
Frequency of monoinfections and coinfections by viral type among infants who tested positive for a respiratory virus (n = 1730). HMPV, human metapneumovirus; HPIV, parainfluenza virus; RSV, respiratory syncytial virus

The frequencies of single infections and coinfections differed among the seven viruses included in this analysis. Monoinfections were most frequent among the 113 infants with influenza (81%), and least likely among the 99 infants with bocavirus (19%) (Figure 1). Bocavirus was detected in only 99 infants and accounted for 5.7% of the infections included in this study. Of the 99 bocavirus cases in this cohort, 81% were detected alongside another viral pathogen, making bocavirus the virus with the highest ratio of coinfected cases to monoinfected cases. Among the 214 cases of RSV, 127 (59%) were monoinfections. Additionally, 51% of HMPV, 55% of HPIV, 77% of HRV, and 56% of CoV were monoinfections.

**Figure 2:**
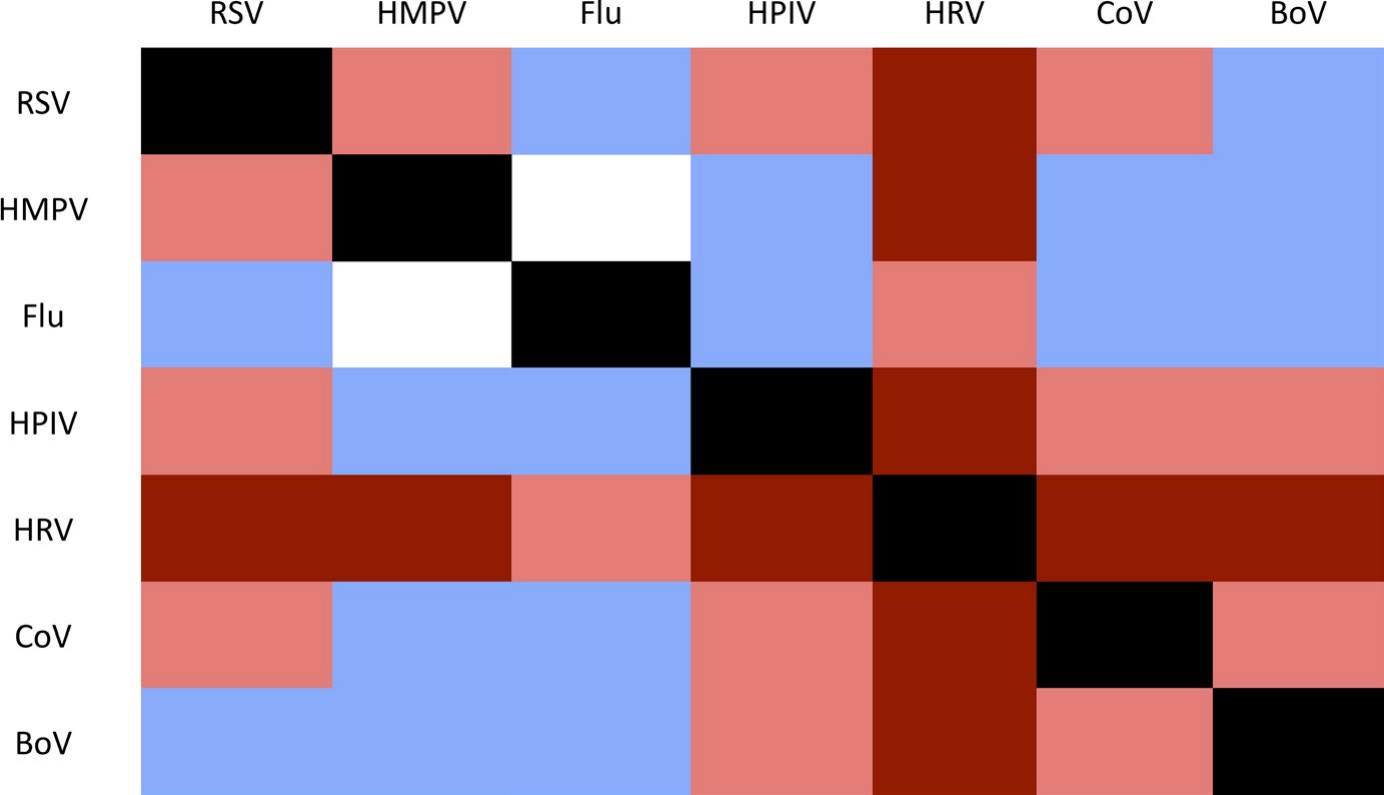
Color map of frequency of viral pairs among 327 cases of coinfection. Dark red indicates > 10% of cases, light red indicates 2%‐10%, light purple indicates < 2%, and white indicates 0 cases. BoV, bocavirus; CoV, coronavirus; Flu, Influenza; HMPV, human metapneumovirus; HPIV, parainfluenza virus; HRV, human rhinovirus; RSV, respiratory syncytial virus

A color plot illustrated the combinations of viruses present among the 327 infants infected with multiple viruses (Figure 2). HRV was most common among all infants with at least one viral infection (68%) and was detected among 95% of coinfection cases. Of the coinfections that did not include HRV, the most common combinations of viruses included RSV, HPIV, and CoV. Besides the combination of influenza and HRV, influenza combined with other viruses each accounted for less than 2% of all cases of coinfection.

Among infants experiencing their first illness episode, 435 (25%) were taken to a care provider for their symptoms, 796 (46%) had symptoms of pneumonia, and 277 (16%) had a fever lasting four days or longer. After adjustment for infant age, LBW, maternal vaccination, and number of children under 5 years old in the household, logistic regression models generated odds ratios for coinfection compared to monoinfection on these three outcomes. Overall, infants with coinfection had 1.4 times the odds of having a fever lasting four days or longer than infants with monoinfection (95% CI 1.1, 2.0; Table 2). Infants with influenza and at least one additional virus were found to have 5.8 times the odds of fever lasting four or more days than infants with influenza alone (95% CI 1.8, 18.7). There was no conclusive evidence for an association between viral coinfection and seeking further medical care (OR 1.1, 95% CI 0.8, 1.5) or pneumonia (OR 1.2, 95% CI 0.96, 1.6). For the subset of coinfected infants with RSV, there was no conclusive evidence for an association of coinfection with seeking care (OR 0.8, 95% CI 0.4, 1.4), pneumonia (OR 1.1, 95% CI 0.6, 2.0), or fever lasting four or more days (OR 0.8, 95% CI 0.4, 1.4). For coinfected infants with influenza, there was a similar lack of evidence for an association of coinfection with seeking care (OR 1.8, 95% CI 0.7, 5.2) or pneumonia (OR 0.6, 95% CI 0.2, 1.8).

**Table 2:** Adjusted odds ratios for associations of coinfection compared to monoinfection on measures of clinical severity, stratified by influenza and RSV

	Sought care			Pneumonia			Fever > 3 days		
	Mono.	Co.	OR^a^ (95% CI)	Mono.	Co.	OR^a^ (95% CI)	Mono.	Co.	OR^a^ (95% CI)	
Overall	347	88	1.1 (0.8, 1.5)	631	163	1.2 (1.0, 1.6)	210	67	**1.4** **(1.1, 2.0)**	
Influenza (n = 113)	24	8	1.8 (0.7, 5.2)	30	7	0.6 (0.2, 1.8)	14	10	**5.8** **(1.8, 18.7)**	
RSV (n = 214)	46	27	0.8 (0.4, 1.4)	77	54	1.1 (0.6, 2.0)	28	18	0.8 (0.4, 1.4)	

Abbreviations: Co., coinfections; Mono., monoinfections; OR, odds ratio; RSV, espiratory syncytial virus. Bold values indicate odds ratios with 95% confidence intervals that do not include the null value of 1.0.

^a^ ORs adjusted for infant age, low birth weight, maternal vaccination receipt, and number of other children under 5 in the household.

Because of the large proportion of cases that involved rhinovirus, a sensitivity analysis was conducted to compare coinfection cases to monoinfection cases without the presence of rhinovirus. Of the 555 cases that did not include rhinovirus, 51 (9.2%) were cases of coinfection, and 504 (90.8%) were monoinfections (Table 3). After excluding rhinovirus, there was little evidence of an association between coinfection with seeking care (OR 0.9, 95% CI 0.4, 1.7), pneumonia (OR 1.1, 95% CI 0.6, 1.9), or having a fever lasting four or more days (OR 1.1, 95% CI 0.5, 2.2). Additional sensitivity analyses were conducted to restrict the dataset to highly pathogenic viruses: after removing bocavirus, rhinovirus, and coronavirus from the dataset, no correlation was found between coinfection and severe disease (see Appendix S1).

**Table 3:** Sensitivity analysis for the adjusted odds ratios and 95% confidence intervals of coinfection compared to monoinfection on three measures of severity, excluding all cases of rhinovirus (HRV)

	Sought care	Pneumonia (n = 263)	Fever > 3 days (n = 106)
Non‐HRV monoinfection (n = 504)	134	238	96
Non‐HRV coinfection (n = 51)	12	25	10
Odds ratios^a^ (95% CI)	0.9 (0.4, 1.7)	1.1 (0.6, 2.0)	1.1 (0.5, 2.2)

^a^ ORs adjusted for infant age, low birth weight, maternal vaccination receipt, and number of other children under 5 in the household.

## 4 DISCUSSION

In this study utilizing prospective community‐based home surveillance for respiratory illness of a birth cohort of infants in rural Nepal, we examined the correlation between respiratory viral coinfection on clinical outcomes. A substantial percentage (19%) of infants who tested positive for a viral respiratory infection had two or more concurrent viruses at their first illness, and infants with coinfection were more likely to experience extended duration of febrile illness than infants with monoinfection.

Our findings confirmed recent reports that coinfection is more prevalent than was believed before routine use of molecular viral diagnostics and that multiple viruses are often present during a respiratory illness episode. Infants with multiple infections tended to be one week older than infants with single infections, but this difference was not statistically significant. In a hospital‐based study in Austria of respiratory viral coinfection among infants, the frequency of viral coinfection compared to monoinfection did not differ by month of age, which aligns with these findings.^2^ This observed age difference may have been due to the effects of certain viruses, such as bocavirus or rhinovirus which are associated with prolonged periods of asymptomatic shedding.^19^

The seven viruses in this analysis differed by their proportion of infections that were monoinfections and those that were coinfections. Bocavirus had a higher ratio of coinfection to monoinfection cases than any other virus in this analysis. Bocavirus has been found to have an extended period of viral shedding after infection, often remaining detectable in young children for over a month after their primary illness event.^19^ Bocavirus is known to often be asymptomatic, so the frequency of multiple infections among infants with bocavirus may be due to infants experiencing symptoms from a subsequent infection and testing positive for a shedding bocavirus. Conversely, influenza had the lowest frequency of coinfection among viruses in our study: Of the 113 infants with influenza documented during their first illness episode, only 19% had one or more additional viruses detected. Influenza infections usually have viral shedding periods for up to seven days, a notably shorter length of time than bocavirus, which may account for part of the difference in coinfection frequency.^14^ The mechanism of infection of the influenza virus may also play a role in its relative adversity to coinfection: The virus has been shown to block the progression of other viruses, and models have illustrated that the influenza virus can rapidly proliferate and generate a robust host inflammatory response, leaving little room for a subsequent viral infection.^14^ As RSV affects the same kind of host cells as influenza, infection with one of the two viruses can prevent infection by the other and may account for the low frequency of influenza alongside other infections among this cohort.^13^ Seasonality of virus circulation did not appear to account for these differences, as multiple pathogens co‐circulated during the period of study.^20^

Rhinovirus was present among 68% of infants experiencing their first respiratory illness episode, making it the most prevalent virus in this cohort (n = 1175). Among the 327 infants with respiratory coinfection, 84% (n = 276) had laboratory‐confirmed rhinovirus alongside at least one additional virus. The prevalence of rhinovirus among infants in this cohort with two or more viruses suggests an ability of rhinovirus to coexist with another viral pathogen, confirming reports of infants with RSV often having coinfection with rhinovirus.^21^ Rhinovirus is usually associated with the common cold, but can lead to a range of symptom severities; rhinovirus can cause pneumonia in infants under 6 months old, but an estimated 15%‐30% of cases are asymptomatic.^22^ The range of clinical presentations associated with rhinovirus monoinfection makes it difficult to determine the source of symptoms in the 276 infants with rhinovirus coinfection.

Disease severity outcomes of pneumonia, seeking further medical care, and fever lasting longer than three days were used because they were measurable attributes of respiratory illness in this population. Fever lasting longer than three days may capture illness episodes that would have resulted in a hospital visit: A United States study found the mean duration of fever for children who were hospitalized to be four days.^23^ Due to the inaccessibility of hospitals in this region, common outcomes of extended hospital stay, admission to an intensive care unit, or requiring supplemental oxygen were unable to be measured. Seeking medical care included visits to local healers and other providers, which is a more accessible form of care for this cohort. This limits the ability to compare these results with hospital‐based studies, but the outcomes used in this analysis better capture the severity of illnesses among infants in this population.

Odds of pneumonia and odds of seeking further medical care did not differ between infants with any monoinfection and those with any coinfection. The subsets of infants with influenza and RSV, respectively, also did not have associations between number of infections and odds of pneumonia or seeking further medical care. This is consistent with findings from hospital‐based studies in South Africa, Japan, Brazil, and Canada, where infants and young children with coinfection did not have increased illness severity compared to those with monoinfection.^7^, ^8^, ^10^, ^24^, ^25^ A 2016 hospital‐based study found RSV coinfection and monoinfection to not differ in disease severity. Those hospitalized with coinfections of RSV and influenza had greater odds of a prolonged stay than RSV alone.^10^ While these reports support the results of this analysis, their hospitalized settings pose a challenge to comparing the data. A 2013 study among childcare attendees reported a similar lack of association between coinfection and illness severity.^26^

Despite not being significantly associated with pneumonia or seeking further care, coinfection was associated with 1.4 times greater odds of having subjective fever for four or more days compared to having a single infection. This combines the effects of multiple types of virus‐virus interaction and may combine pairings that lengthen fever duration and pairings that shorten it. However, the net association between coinfection and extended fever among infants indicated that the interaction of multiple viruses did influence the clinical progression of respiratory infection. Analyzing the subset of infants who tested positive for influenza showed a stronger association between multiple infections and duration of fever: Those with influenza alongside a second or third virus had 5.8 times the odds of fever lasting four days or more than infants with influenza alone. Despite the low number of influenza cases and the subsequently wide confidence interval, this study provided strong evidence that coinfection with influenza was associated with greater odds of prolonged fever than influenza alone. This finding confirms the conclusion of other studies conducted in urban settings that infants with influenza can experience longer duration of illness when one or more other viruses are present,^2^, ^11^, ^26^ although a hospitalized study in Spain reported no association between coinfection and illness duration.^21^ This discrepancy may be due to differences in viral pairings, or the ages of the study population.

To determine the effects of coinfection that did not include rhinovirus, a sensitivity analysis evaluated the associations between coinfection and symptom severity outcomes in the subset of infants who did not test positive for rhinovirus. Of the 555 infants without rhinovirus, coinfection was not associated with seeking further care, pneumonia, or fever lasting for four or more days compared to monoinfection. This lack of association between coinfection and fever after the exclusion of rhinovirus indicates that rhinovirus plays a significant role in the clinical severity of coinfection cases, and it should be included in considerations of the interactions between multiple viruses.

There are several limitations of this study. These data were taken from weekly household visits, so the timeline of infection is unclear among infants who tested positive for multiple viruses in the same week. As the interaction between two viruses may differ by the order in which they infect, weekly data may combine the effects of multiple types of interactions. Infant symptom data were collected by weekly subjective report from the mother, which limits the objectivity of our findings. Our definition of coinfection was limited by the seven respiratory viruses included in this analysis. Additionally, despite the broad scale of the original trial and the large cohort of infants followed in this study, the numbers of cases in each specific virus pairing was too small to analyze the effects of each potential combination of viruses individually. As the clinical effects of multiple infections may differ by the identity of each virus, evaluating each potential pair of viruses would best capture the relative severity of coinfections, but would require a sample size difficult to achieve in single center trials. Analyzing subsets of infants with influenza and RSV allowed for more specific investigation of the effects of coinfection on clinical severity in this cohort. Finally, this study does not speak to differences in presentation between repeated cases of coinfection. A recent analysis from this cohort found that the clinical presentations of multiple episodes of respiratory viral infection are comparable.^20^

This analysis of infants experiencing their first respiratory illness demonstrated that coinfection is associated with increased duration of febrile illness, especially among infants with influenza. While seeking care and having symptoms of pneumonia did not notably differ between infants with monoinfections and infants with coinfections, extended fever duration is a serious concern for rural communities where respiratory illness is a leading cause of infant mortality. This analysis demonstrates that coinfection affects clinical outcomes in a cohort of infants in a rural setting. On an individual level, these findings demonstrate the importance of diagnostic methods that can accurately identify coinfection, which may play an important role in clinical outcomes. On a broader scale, vaccines for influenza, RSV, and other respiratory illnesses are being considered internationally, and the primary trial results showed maternal influenza immunization to effectively protect infants from respiratory illness.^14^ As vaccines for respiratory viruses continue to be developed and tested around the world, coinfection remains an important clinical factor that needs to be considered when designing and measuring the effects of these interventions.

## AUTHOR CONTRIBUTION

**Anne Emanuels:** Conceptualization (equal); Formal analysis (lead); Validation (equal); Writing‐original draft (lead). **Stephen E. Hawes:** Conceptualization (equal); Formal analysis (supporting); Project administration (equal); Supervision (equal); Validation (equal). **Kira L. Newman:** Conceptualization (supporting); Formal analysis (supporting); Validation (equal). **Emily T. Martin:** Conceptualization (supporting); Methodology (equal); Supervision (supporting); Writing‐review & editing (supporting). **Janet Englund:** Project administration (supporting); Supervision (supporting); Validation (supporting); Writing‐review & editing (equal). **James M. Tielsch:** Investigation (equal); Project administration (equal); Writing‐review & editing (supporting). **Jane Kuypers:** Investigation (equal); Project administration (supporting); Writing‐review & editing (supporting). **Subarna Khatry:** Investigation (equal); Writing‐review & editing (supporting). **Steven C. LeClerq:** Investigation (equal); Writing‐review & editing (supporting). **Joanne Katz:** Investigation (equal); Writing‐review & editing (supporting). **Helen Chu:** Conceptualization (equal); Investigation (equal); Project administration (equal); Supervision (lead); Writing‐review & editing (equal).

## Supporting information

Supplementary Material
