# Supplementary material for: Respiratory viral coinfection in a birth cohort of infants in rural Nepal
Source: Influenza Other Respir Viruses. 2020 Jun 22;14(6):739–46. doi: 10.1111/irv.12775 (PMC7578290; doi:10.1111/irv.12775)
Supplement: Supplementary file 1 — Supplementary Material [file IRV-14-739-s001.docx]

**Supporting Information**

**Table A.**

|  | Fever >=3 Days  (n=496) | Fever >= 4 Days  (n=277) | Fever >= 5 Days  (n=153) |
| --- | --- | --- | --- |
| Monoinfection | 392 | 210 | 112 |
| Coinfection | 104 | 67 | 41 |
| Odds Ratios (95% CI) | 1.11 (0.85, 1.46) | 1.45 (1.06, 1.98) | 1.64 (1.12, 2.42) |

**Table B.**

|  | Sought Care | Pneumonia | Fever >= 4 Days |
| --- | --- | --- | --- |
| Monoinfection (n=590) | 167 | 285 | 122 |
| Coinfection(n=24) | 7 | 16 | 7 |
| Odds Ratios (95% CI) | 1.05 (0.43, 2.58) | 2.11 (0.88, 5.05) | 1.66 (0.67, 4.13) |

**Table C.**

|  |  | Clinical Symptoms, n (%) | | | | |
| --- | --- | --- | --- | --- | --- | --- |
| Virus Detected | **Infection Type** | **Fever** | **Cough** | **Wheeze** | **Difficulty Breathing** | **Draining Ear** |
| RSV | Monoinfection (n=127) | 84 (66) | 108 (85) | 74 (58) | 70 (55) | 6 (5) |
|  | Coinfection (n=87) | 50 (57) | 77 (89) | 53 (61) | 45 (52) | 11 (13) |
| HMPV | Monoinfection (n=54) | 33 (61) | 41 (76) | 32 (59) | 32 (59) | 3 (6) |
|  | Coinfection (n=44) | 22 (50) | 35 (80) | 28 (64) | 23 (52) | 4 (9) |
| Influenza | Monoinfection (n=90) | 69 (77) | 42 (47) | 33 (37) | 34 (38) | 7 (8) |
|  | Coinfection (n=22) | 17 (77) | 14 (64) | 6 (27) | 8 (36) | 0 (0) |
| HPIV | Monoinfection (n=113) | 68 (60) | 74 (65) | 52 (46) | 48 (42) | 2 (2) |
|  | Coinfection (n=91) | 59 (65) | 69 (76) | 52 (57) | 42 (46) | 4 (4) |
| Rhinovirus | Monoinfection (n=902) | 414 (46) | 555 (62) | 419 (46) | 377 (42) | 48 (5) |
|  | Coinfection (n=270) | 152 (56) | 211 (78) | 146 (54) | 126 (47) | 19 (7) |
| Coronavirus | Monoinfection (n=101) | 54 (53) | 75 (74) | 44 (44) | 39 (39) | 5 (5) |
|  | Coinfection (n=75) | 38 (51) | 57 (76) | 41 (55) | 34 (45) | 4 (5) |
| Bocavirus | Monoinfection (n=19) | 11 (58) | 9 (47) | 13 (68) | 11 (58) | 0 (0) |
|  | Coinfection (n=80) | 48 (60) | 60 (75) | 39 (49) | 30 (38) | 2 (3) |
